# Mimivirus reveals Mre11/Rad50 fusion proteins with a sporadic distribution in eukaryotes, bacteria, viruses and plasmids

**DOI:** 10.1186/1743-422X-8-427

**Published:** 2011-09-07

**Authors:** Takashi Yoshida, Jean-Michel Claverie, Hiroyuki Ogata

**Affiliations:** 1 Laboratory of Marine Microbiology, Graduate School of Agriculture, Kyoto University, Kyoto 606-8502, Japan; 2 Structural and Genomic Information Laboratory, CNRS-UPR 2589, Aix-Marseille University, Mediterranean Institute of Microbiology, 163 Avenue de Luminy, Case 934, 13288 Marseille Cedex 9, France

**Keywords:** Fusion genes, Viruses, Mimivirus, Viral gene pool, DNA repair, Replication, SbcD/SbcC

## Abstract

**Background:**

The Mre11/Rad50 complex and the homologous SbcD/SbcC complex in bacteria play crucial roles in the metabolism of DNA double-strand breaks, including DNA repair, genome replication, homologous recombination and non-homologous end-joining in cellular life forms and viruses. Here we investigated the amino acid sequence of the Mimivirus R555 gene product, originally annotated as a Rad50 homolog, and later shown to have close homologs in marine microbial metagenomes.

**Results:**

Our bioinformatics analysis revealed that R555 protein sequence is constituted from the fusion of an N-terminal Mre11-like domain with a C-terminal Rad50-like domain. A systematic database search revealed twelve additional cases of Mre11/Rad50 (or SbcD/SbcC) fusions in a wide variety of unrelated organisms including unicellular and multicellular eukaryotes, the megaplasmid of a bacterium associated to deep-sea hydrothermal vents (*Deferribacter desulfuricans*) and the plasmid of *Clostridium kluyveri*. We also showed that R555 homologs are abundant in the metagenomes from different aquatic environments and that they most likely belong to aquatic viruses. The observed phyletic distribution of these fusion proteins suggests their recurrent creation and lateral gene transfers across organisms.

**Conclusions:**

The existence of the fused version of protein sequences is consistent with known functional interactions between Mre11 and Rad50, and the gene fusion probably enhanced the opportunity for lateral transfer. The abundance of the Mre11/Rad50 fusion genes in viral metagenomes and their sporadic phyletic distribution in cellular organisms suggest that viruses, plasmids and transposons played a crucial role in the formation of the fusion proteins and their propagation into cellular genomes.

## Background

DNA double-strand breaks (DSBs) are a major cause of genomic instability and can lead to chromosomal aberration and cancer [1,2]. DSBs occur in intermediate steps during normal DNA metabolic processes such as genome replication, meiotic recombination and programmed DNA rearrangement, but are also caused by DNA-damaging agents, including ionizing radiation as well as genotoxic chemicals. All cellular organisms possess a set of conserved proteins to cope with this dangerous form of genomic DNA; the Mre11/Rad50 complex in eukaryotes/archaea and its bacterial homologs (the SbcD/SbcC system) are the key players of the DSB metabolism generating a recombinogenic 3' overhang [3]. Due to their ubiquitous presence in cellular organisms, it has been suggested that the last universal common ancestor (LUCA) already possessed this system [4]. Mre11 and SbcD are nucleases belonging to the calcineurin-like phosphoesterase family. Rad50 and SbcC are ABC ATPases belonging to the Structural Maintenance of Chromosomes (SMC) superfamily, and exhibit a long coiled-coil domain (~500Å when fully stretched) used to bridge two DNA molecules. The *Escherichia coli *SbcD/SbcC complex has an affinity for DNA hairpins and is known to generate DSBs at these sites [2,5]. Homologous enzymes are also found in viruses; for instance, T4 phage encodes a Rad50 homolog (gp46) and a Mre11 homolog (gp47). These proteins are involved in the recombination-dependent DNA replication, an elegant solution to the end replication problem of linear viral genomes [6,7]. In bacteria, archaea and T4, these nuclease and ATPase are encoded in an operon [2,8].

Mimivirus (Acanthamoeba polyphaga mimivirus; APMV), a giant dsDNA virus infecting *Acanthamoeba *spp., is the prototype of the Mimiviridae family, the latest addition to the nucleocytoplasmic large DNA virus (NCLDV) group. Mimivirus is the largest among known viruses in both particle size (~750 nm) and genome length (1.2 Mb-genome encoding 1018 genes) [9-11]. The Mimivirus genome encodes at least eight putative DNA repair enzymes capable of correcting mismatches or errors induced by oxidation, UV irradiation and alkylating agents, including the recently analyzed putative mismatch repair enzyme MutS7 (L359) that exhibits a distinctive domain organization shared by other giant viruses [12]. Mimivirus R555 originally annotated as a Rad50 homolog [10] is part of this uniquely complete Mimivirus DNA repair tool box. The R555 gene product specifically attracted our attention following a study that pointed out the existence of numerous homologs closely related to R555 in a marine metagenomic data set [13]. Monier *et al*. analyzed the sequences gathered by the Global Ocean Sampling (GOS) Expedition [14], and identified 5,293 homologs for 229 Mimivirus proteins in the metagenomic data set (mostly 0.1-0.8 μm size fractions). The number of such homologs for each of the Mimivirus proteins was variable ranging from 1 to 249. R555 homologs were found to be the most abundant with 249 GOS scaffold matches, followed by 189 matches for R382 (mRNA capping enzyme) and 185 matches for R322 (B-type DNA polymerase). Here, we analyzed the sequence of R555 using database searches and phylogenetic reconstruction, and assessed the abundance of its close homologs in another large metagenomic data set generated by the Metagenomic Profiling of Nine Biomes (BIOME), which consists of 42 viral (<0.22 μm size fractions with a concentration of viral DNAs) and 45 microbial metagenomes (typically >0.22 μm size fractions) [15].

## Results

### R555 encodes a fusion protein with Mre11-like and Rad50-like domains

**Figure 1:**
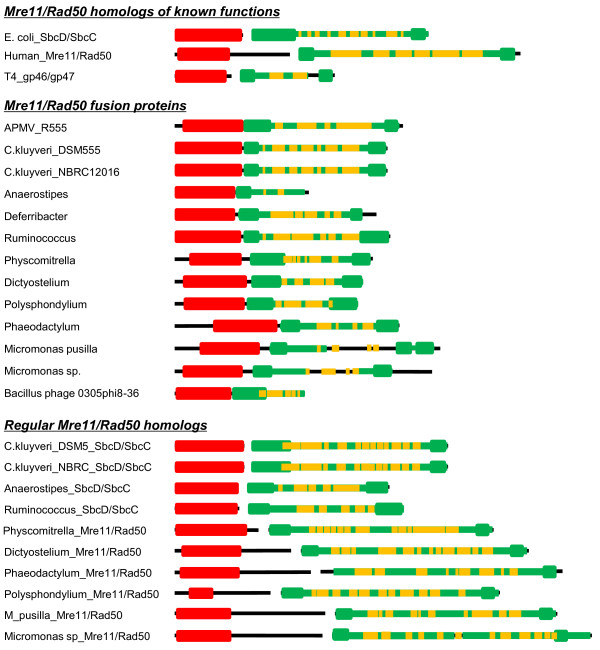
**Domain organization of Mre11/Rad50 (SbcD/SbcC) homologs**. Sequence regions corresponding to nuclease (SbcD/COG0420) and ATPase (SbcC/COG0419) are highlighted by red and green lines, respectively. Within SbcC regions, ATP-binding domains (cd03279) are indicated by thicker green lines, and the predicted coiled-coil regions are indicated by orange line. This diagram is approximately drawn to scale. Database entries for sequences are as follows: Ecoli_SbcD/SbcC, *Escherichia coli *K12 (NP_414932/NP_414931); Human_Mre11/Rad50, *Homo sapiens *(NP_013951/NP_014149); T4_gp46/gp47, *E. coli *phage T4 (NP_049672/NP_049669); C.kluyveri_DSM_SbcD/SbcC, *Clostridium kluyveri *DSM 555 (YP_001393540/YP_001393539); C.kluyveri_NBRC_SbcD/SbcC, *Clostridium kluyveri *NBRC12016 (B9DY24_CLOK1/B9DY23_CLOK1); Anaerostipes_SbcD/SbcC, *Anaerostipes caccae *DSM 14662 (ZP_02418331/ZP_02418330); Ruminococcus_SbcD/SbcC, *Ruminococcus *sp. 5_1_39BFAA (ZP_04855442/ZP_04855441); Physcomitrella_Mre11/Rad50, *Physcomitrella patens *subsp. patens. (XP_001755538/Pp1s51_220V6); Dictyostelium_Mre11/Rad50, *Dictyostelium discoideum *(XP_629462/XP_628997); Phaeodactylum_Mre11/Rad50, *Phaeodactylum tricornutum *CCAP 1055/1 (XP_002181412/XP_002179845); Polysphondylium_ Mre11/Rad50, *Polysphondylium pallidum *PN500 (EFA85561/EFA85515); M_pusilla_Mre11/Rad50, *Micromonas pusilla *CCMP1545 (XP_003063605/XP_003055898); Micromonas sp_Mre11/Rad50, *Micromonas *sp. RCC299 (XP_002504614/XP_002507736).

ORF R555 (1351 aa) of Mimivirus was initially annotated as a putative DNA repair protein [10]. When searched against the NCBI non-redundant protein sequence database using BLAST, R555 showed significant sequence similarities to 31 bacterial SbcC DNA repair protein sequences (E-value: 10^-4^~ 10^-9^), which were aligned with the C-terminal part of R555. Similar to bacterial SbcC (COG0419), the corresponding domain of R555 showed a set of ATP-binding motifs: an N-terminal Walker A motif (P-loop) and a C-terminal Walker B motif followed by a D-loop motif [16]. A structural analysis of its Rad50 archaeal homolog in *Pyrococcus furiosus *suggests that two catalytic domains are build up by the ATP-dependent dimerization of these ATPase segments in an anti-parallel orientation [16]. In addition to the ATPase domain, an analysis of the conserved domain [17] revealed the presence of a Metallophos domain (pfam00149; calcineurin-like phosfatase family) [18] in the N-terminal region of R555 [19] (Figure 1). The Metallophos protein family includes a diverse range of phosphoesterases as well as members of DNA repair exonucleases (COG0420), such as yeast Mre11 and bacterial SbcD proteins [18]. When the N-terminal region of R555 (residues 1-250) was searched against the database by BLAST, the partial sequence was found most similar to the *Thermus thermophilus *HB8 SbcD homolog (E-value = 10^-7^). Members of the SbcD family proteins (COG0420) exhibit a conserved sequence DXH(X_25_)GDXXD(X_25_)GNHD/E near the metal chelating site [18,20,21]. This motif was found in R555 except for the third aspartic acid residue that was replaced by histidine. Several residues in the C-terminal part of *P. furiosus *Mre11 were reported to be involved in the nuclease activity (His173, His206 and His208 in *P. furiosus *Mre11) [21,22]. The R555 sequence possesses all of these catalytic residues except for an aspartic acid residue (Asp242) replacing the His206 of *P. furiosus *Mre11. In conclusion, the Mimivirus R555 gene product corresponds to the fusion of an N-terminal Mre11-like domain with a C-terminal Rad50-like domain.

### Identification of similar fusion proteins in viruses, plasmids, bacteria and eukaryotes

To search for additional instances of Mre11/Rad50 (or SbcD/SbcC) fusion proteins, we performed PSI-BLAST searches against the UniProt database using position specific scoring profiles corresponding to COG0419 (SbcC) and COG0420 (SbcD) as queries. We successfully found twelve sequences showing significant similarities to both profiles from bacteria, eukaryotes and a phage (Figure 1). Five bacteria (four firmicutes and one thermophilic bacterium of the phylum Deferribacteres) were found to possess an SbcDC fusion: the anaerobic soil bacteria *Clostridium kluyveri *DSM555 (A5F9P1_CLOK5) and *C. kluyveri *NBRC 12016 (B9E6H1_CLOK1), the colon bacteria *Anaerostipes caccae *DSM 14662 (B0MG68_9FIRM), the rumen-associated *Ruminococcus *sp. 5_1_39BFAA (C6JB59_9FIRM), and *Deferribacter desulfuricans *SSM1 (D3PEM5_9BACT), a thermophilic sulfur reducing bacterium isolated from a deep-sea hydrothermal vent. The eukaryotes exhibiting a fused version of protein sequence were the moss *Physcomitrella patens *subsp. *patens *(A9RK34_PHYPA), the slime molds *Dictyostelium discoideum *(Q8T663_DICDI) and *Polysphondylium pallidum *PN500 (D3AVR3_POLPA), the marine pennate diatom *Phaeodactylum tricornutum *CCAP 1055/1 (B7FXD6_PHATR), and the photosynthetic Prasinophyceae *Micromonas pusilla *CCMP1545 (C1N0B1_9CHLO) and *Micromonas *sp. RCC299 (C1FJU6_9CHLO), found throughout diverse marine environments. Finally, a similar fusion protein was also found in the *Bacillus *phage 0305phi8-36 (A7KV18_9CAUD). In both strains of *C. kluyveri*, the fusion proteins were encoded in their plasmids. In *Deferribacter*, the gene for the fusion protein was flanked by two sets of transposases (IS*200*/IS*605*) on the 308-kb megaplasmid. In the cases of *Anaerostipes *and the *Bacillus *phage, one of the P-loop NTPase segments normally part of SbcC was not observed. Mimivirus and the the *Bacillus *phage encode only the fused version of homologs. However, in all of the cellular organisms exhibiting a fused form of gene except for *Deferribacter*, a normal set of genes separately encoding Mre11/Rad50 or SbcD/SbcC homologs was identified (Figure 1), suggesting that the role of the fused version of proteins in these cellular organisms may differ from that of the regular Mre11/Rad50 (SbcD/SbcC) complexes.

### The fusion form is compatible with known interactions observed in the Mre11/Rad50 complex

Structural analysis of the *Thermotoga maritima *Mre11/Rad50 complex revealed that Mre11 comprises a phosphodiesterase domain, an accessory DNA-binding "capping" domain and the most C-terminal helix-loop-helix (HLH) domain, and that the HLH domain is involved in the direct interaction with the root of the Rad50 coiled-coil domain (additional file 1) [1]. Our sequence analysis shows that most of the newly identified fusion proteins exhibit a sequence region that is similar in length to the sequences of the Mre11 capping and HLH domains, except for the fusion protein of *Bacillus *phage lacking this part of sequence. Furthermore, three residues in the HLH domain previously suggested to be responsible for the interaction with Rad50 were found to be relatively well conserved in the fusion protein sequences (additional file 1). Finally, the fusion of Mre11 and Rad50 in this order from the N-terminus appears compatible with the structural organization of the *T. maritima *Mre11/Rad50 complex, as the C-terminal end of Mre11 is located in a close proximity (~28Å) to the N-terminus of Rad50. Such a distance would correspond to a linker of nine to twelve residues in an approximately extended conformation. The sequences of R555 and most of the newly identified fusion proteins appear to possess such extra residues between the Mre11-like and Rad50-like regions. This result suggests that the inter-domain interactions within the protein encoded by the fusion gene might mimic those observed in the *T. maritima *Mre11/Rad50 complex.

### Fused versions of protein sequences are distinct from the canonical Mre11/Rad50 or SbcD/SbcC sequences

**Figure 2:**
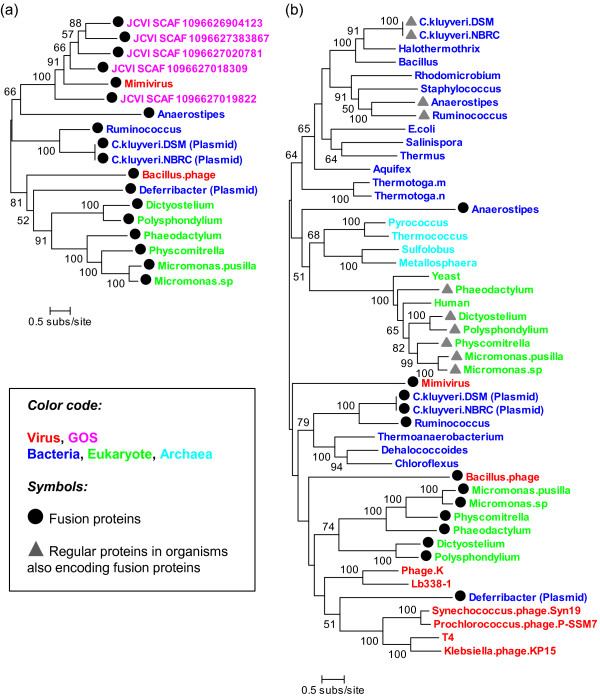
**Maximum likelihood tree of Mre11/Rad50 homologs**. (a) A phylogenetic tree for the fused version of Mre11/Rad50 homologs including the Mimivirus R555 homologs from the GOS data. (b) A phylogenetic tree including non-fused versions of Mre11/Rad50 (and SbcD/SbcC) homologs. The trees are midpoint. Bootstrap values below 50% are not shown. Database entries for sequences are show in additional file 2.

We first performed a phylogenetic tree reconstruction of the Mre11/Rad50-like fusion protein sequences, including several GOS sequences showing a strong similarity to R555. No R555 homologs from the GOS data set could be fully aligned with R555 due to the short size of the metagenomic scaffolds. However, five GOS sequences were found to contain an entire Mre11 domain and part of the Rad50 domain, and were included in this analysis. In the resulting tree (Figure 2(a) and additional file 2), R555 and its GOS homologs formed a monophyletic group supported by a bootstrap value of 100%, confirming the accuracy of our BLAST procedure used to detect close homologs of R555 in the metagenomes. Eukaryotic sequences ("the moss-diatom-prasinophyte group") and three sequences from closely related bacteria ("the *Clostridium-Ruminococcus *group") showed well supported groups (bootstrap values were 91% and 100%, respectively). However, other bacterial sequences were found scattered in the tree. Sequences from plasmids or viruses did not show any particular grouping.

A more comprehensive evolutionary picture emerged when regular (i.e., "non-fused") versions of sequences were included in our phylogenetic analysis (Figure 2(b)). In this phylogenetic tree reconstruction, regular protein sequences were concatenated to obtain a multiple alignment with the fused protein sequences. In the resulting tree, the classical regular sequence versions showed groupings corresponding to the three domains of life: a bacterial clade represented by *E. coli *SbcD/SbcC, an archaeal Mre11/Rad50 clade, and a eukaryotic clade represented by the yeast and human Mre11/Rad50 proteins. These regular sequences forming three clades congruent with the three domains are hereafter referred to as the "canonical" Mre11/Rad50 (or SbcD/SbcC) homologs.

Remarkably, the thirteen fused versions of protein sequences were placed outside the three canonical clades, indicating that they are only distantly related to the experimentally characterized classical versions of Mre11/Rad50 homologs that are widespread within the cellular organisms. Furthermore, the fused sequences did not make a monophyletic group of their own, showing no specific affinity with each other. Non-fused and non-canonical versions of sequences from several bacteria (*Thermoanaerobacterium*, *Dhalococcoides*, *Chloroflexus*) and phages were found intercalated among the branches leading to the fused protein sequences. As previously described, we identified non-fused versions of Mre11/Rad50 or SbcD/SbcC homologs in ten cellular organisms harboring also a fused version of gene (Figure 1). These non-fused versions of proteins were clearly placed within the canonical clades, suggesting that the fused versions of genes in these bacteria and eukaryotes are not derived from the regular counterparts in the same cellular genome.

### R555 homologs from the GOS and BIOME metagenomes originate in viruses

**Figure 3:**
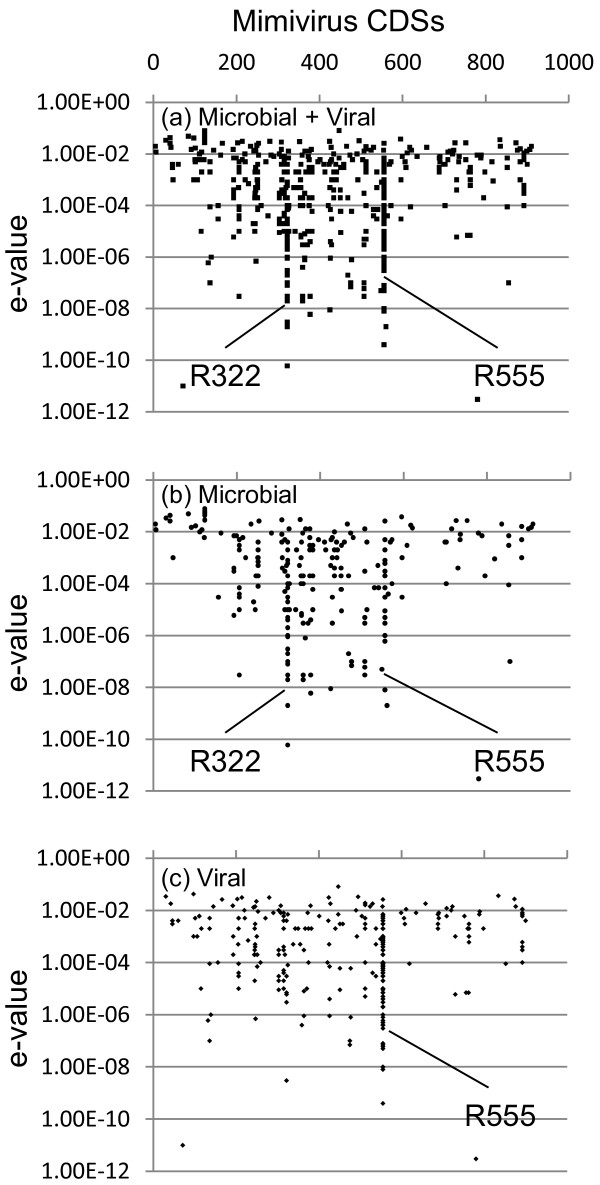
**Distribution of Mimivirus CDS homologs in the BIOME metagenomic data set**. (a) 728 sequences identified in the entire BIOME data set. (b) 467 sequences from viral fractions. (c) 261 sequences from microbial fractions.

To investigate further the taxonomic origin of the 249 R555 homologs from the GOS metagenomic data set, additional ORFs from these R555 homolog-harboring scaffolds [13] were searched against the UniProt database using BLAST. 85 ORFs from 68 GOS-scaffolds showed significant similarities (E-value<10^-5^) to database sequences. Notably, 50 of them were most similar to viral sequences (49 phage genes and one eukaryotic virus sequence), and 40 of the 68 scaffolds (60%) were found to contain viral-like genes in addition to the Mimivirus R555 homolog (additional file 3 and 4). Phage protein homologs found in these scaffolds included primases (ORF26 of *Staphylococcus *phageG1, ORF023 of *Staphylococcus *phage Twort, gp21.95 of *Bacillus *phage SPO1, gp068 of *Lactobacillus *phage LP65, p152 of *Synechococcus *phage S-PM2, gp048 of *Listeria *phage A511) and endonucleases (gp3 of *Yersinia *phage Yepe2, gp14 of Enterobacteria phage K1F, gp3 of *Salmonella *phage phiSG-JL2). On one hand, this result suggests that many of these GOS scaffolds were of viral origins. On the other hand, the abundance of ORFs most similar to phage genes suggests that the R555 homologs in the GOS data set do not necessarily correspond to large DNA viruses but might belong to smaller viruses. To further investigate this possibility, we examined the distribution of R555 homologs within another large metagenomic data set (i.e. BIOME) comprising 87 metagenomes (42 from viral fractions and 45 from microbial fractions) from nine environments (animal, coral, fish, insect, marine, fresh water, hyper-saline, microbialite, subterranean) [15]. By the same two-way BLAST approach used to detect Mimivirus-like sequences in the GOS data set [13], we identified 728 BIOME reads (with average length of 150 bp) similar to Mimivirus protein sequences (for 184 distinct Mimivirus genes). Two hundred sixty-one sequences were from microbial fractions (> 0.22 μm), while 467 originated from viral fractions (0.22 μm-filtrate purified by cesium chloride (CsCl) step gradients). The relative abundance of the Mimivirus-like sequences were high in marine (viral), fresh water (microbial/viral), hyper-saline (viral) and microbialite (microbial/viral) environments (additional file 5). Interestingly, of the 184 Mimivirus protein sequences with close homologs in the BIOME data set, R555 exhibited an exceptionally large number of homologs (288 BIOME reads), followed by much smaller numbers for R322 (B-type DNA polymerase) with 38 reads, and L123 (hypothetical protein) with 20 reads (Figure 3). Furthermore, 267 of the 288 (93%) R555 homologs were found in the viral fractions of the BIOME data. Overall, R555 homologs are consistently abundant in different environmental sequence data sets, and enriched in viral fractions (< 0.22 μm) in the case of the BIOME data. Such an enrichment in viral size fractions was not observed for R322 (B type DNA polymerase), the gene most often used as a reference to monitor the presence of large DNA viruses. This discrepancy between the R555 and DNA polymerase read counts suggests that some of previously identified R555-like homologs in the GOS data set might in fact originate from phages rather than from large eukaryotic viruses. It should be noticed that the Mre11/Rad50 protein fusion in environmental sequence data was only confirmed for a few cases of long sequences in the GOS data set, although both of the nuclease and ATPase domains of R555 showed many closely related sequences in the BIOME data (data not shown).

## Discussion

While investigating the DNA repair functions encoded by the Mimivirus genome, we discovered that the R555 initially annotated as a Rad50 homolog was in fact a fusion between Mre11 and Rad50, two proteins known to be involved in the repair of DNA double-strand breaks. Using the R555 sequence as a template, we then found that similar fusion proteins are present in a wide variety of unrelated organisms: phage, bacteria, unicellular and multicellular eukaryotes, albeit with a highly sporadic distribution. To our knowledge, this is the first description of the existence fusion genes encoding both Mre11-like and Rad50-like domains.

Interestingly, some of the fusion genes were identified in plasmids (*Clostridium kluyveri *and *Deferribacter*). In the case of *Deferribacter*, the plasmid-encoded *sbcDC *fusion gene was found flanked by insertion sequences (i.e., transposons). Our analysis of various metagenomic data sets revealed that close homologs of R555 are abundant in different aquatic environments and that they are most likely associated with viruses. Finally, our phylogenetic analysis indicated that the Mre11/Rad50 (or SbcDC) fusion proteins are only distantly related to the canonical versions of homologs and do not form a monophyletic group. No simple evolutionary scenario explains these observations. A possibility is that the cellular fusion genes were vertically derived from an ancestral gene present as a paralog of the canonical genes in an ancestral cellular organism such as LUCA. These cellular non-canonical genes were then lost from most cellular lineages but were recruited by viruses including the ancestor of Mimivirus. However, we consider this "cell-central" hypothesis unlikely as this scenario postulates numerous independent gene loss events in cellular organisms. A more likely explanation is to assume the presence of non-canonical genes in ancestral viruses and the occurrence of multiple gene fusion events.

In general, gene fusions are expected to facilitate or simplify the co-expression and assembly of protein domains initially encoded in separate genes. Such a physical link of two associated functions at the genomic level also enhances the probability of successful lateral gene transfers. Known examples of fused proteins in viruses include the primase and helicase domain fusions in large DNA viruses [23]. More recently, the mismatch repair protein MutS of Mimivirus was suggested to combine functions normally encoded in separate proteins, thanks to the fusion of the classical mismatch recognition domains with a nicking endonuclease domain [12]. The fusion proteins of this type, now classified in the MutS7 subfamily, are abundant in large DNA viruses as well as in environmental metagenomes, but also present in a few distantly related cellular organisms (i.e., the mitochondria of octocorals and Epsilonproteobacteria). Our observations on the Mre11/Rad50 fusion proteins show an intriguing resemblance to those made on the MutS7 subfamily.

The apparent contradiction between their abundance in metagenomes and the sporadic distribution in unrelated (but mostly marine) cellular organisms suggest that the true niche of these protein variants is in viruses. Viruses are abundant in aquatic environments, are known to hold a huge genetic diversity yet underrepresented in the current databases, and are suggested to be the place of the creation of new genes [24]. We propose that viruses, plasmids and/or transposons might have played a key role in the emergence of these Mre11/Rad50-like fusion proteins as well as in their subsequent propagation into different cellular organisms. The non-fused and non-canonical versions of Mre11/Rad50 homologs from viruses (such as gp46/gp47 of T4 and other marine T4-like viruses) were found outside the canonical clades corresponding to the three domains of life. We confirmed that almost all T4-like viruses with complete genomes possess homologs of these genes (data not shown). This phylogenetic feature is consistent with our hypothesis that viruses are the evolutionary origin of the Mre11/Rad50 fusion proteins found in bacteria and eukaryotes.

Gene fusions probably occurred several times in different viral lineages using an operon structure as an evolutionary template towards fused genes. Lateral transfers then possibly spread the fused genes into different viruses and cellular organisms. For instance, the presence of close homologs in mosses (Streptophyta), diatoms (stramenopiles) and green algae (Chlorophyta) suggests gene transfers among these very distantly related eukaryotes via unidentified intermediates. Forterre hypothesized that the cellular DNA informational proteins have been recruited independently in the three domains of life from different viruses, which shared a few common DNA processing enzymes such as the canonical Rad50/Mre11 [25]. In our phylogenetic tree (Figure 2(b)), cellular non-canonical versions (in either fused or non-fused forms) were found intercalated among the branches leading to non-canonical versions of viral proteins. It is possible to see this branching pattern as another (but more recent) case of the lateral flow of DNA processing genes from the virus gene pool to cellular genomes. Future efforts in generating much longer and deeper metagenomic reads are needed to better understand the evolution of these new types of proteins.

**Figure 4:**
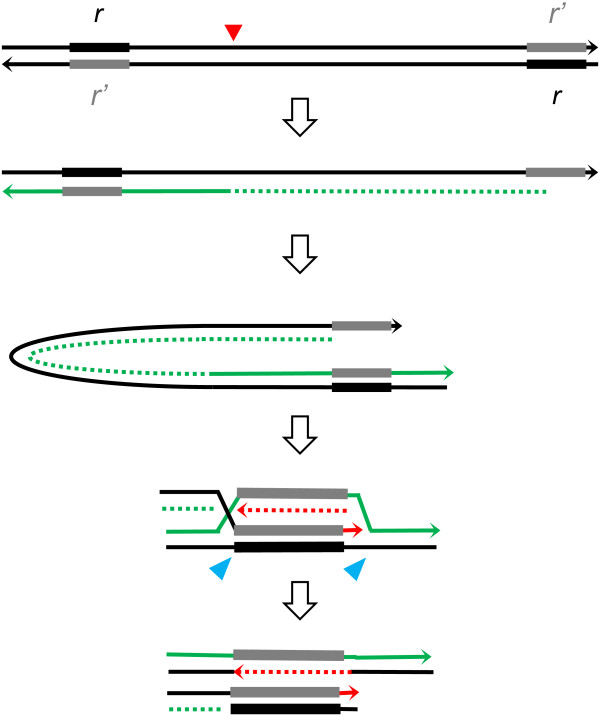
**Hypothetical model for the end replication of the linear Mimivirus genome**. The Mimvirus genome is illustrated by two black lines with arrowheads indicating the 3'-ends. Inverted repeats are indicated by black (forward) and gray (reverse) bars. Red triangle indicates the position of the putative origin of replication corresponding to the 400,000 nt position, where gene excess and A+C excess curves show a reversal. Green sold lines indicate newly synthesized leading strands, and green dotted lines newly synthesized lagging strands. The R555 gene product is hypothesized to be involved in the DSB resection. 3' ss end then invades the homologous duplex at the opposite extremity of the genome, and the DNA synthesis starts (solid red line for leading and dotted red line for lagging strand synthesis). Cross-strand structure is resolved by cleavages (blue triangles).

The function of R555 in the replication cycle of Mimivirus is currently unknown. In a previous work [11], R555 was found to have the same intermediary expression pattern as other genes involved in DNA replication. R555 may thus be involved in repair of transient DSBs, for instance, in the region ahead of the advancing replication fork, since the Mimivirus genome encodes a topoisomerase II (R480) [10], which removes superhelical tension by introducing transient DSBs [26]. Alternatively, the function of R555 may be associated with genomic properties more specific to Mimivirus. As mentioned earlier, the T4 proteins gp46 and gp47 (homologs of SbcC/SbcD, respectively) are involved in the recombination-dependent DNA replication (RDR) of the viral genome [6]. T4 particles carry a linear dsDNA molecule. The replication of such a linear molecule has to overcome the problem of synthesizing the complementary strand of the 3' terminus of the template DNA. T4 solves this problem using RDR linked to its terminally redundant and circularly permuted linear genome. In essence, 3' single stranded (ss) end invades homologous duplex DNA near the opposite end of the chromosome (either the same DNA molecule or its sister). gp46/gp47 appears to contribute to the generation of the 3' ss end (by a process called "resection") as well as to the bridging of two DNA ends. There is also evidence suggesting that poxviruses uses homologous recombination and DSB repair for full-size genome replication, where the G5 protein (a FEN1-like homolog) plays a crucial role [27]. The Mimivirus R555 may be involved in a similar process for the solution of the end replication problem. The chromosome of Mimivirus is not circular permuted, but exhibits a quasi-perfect (616/617 bp) inverted repetition of a 617-bp sequence near its termini [28]. These homologous regions may serve as a template to complete the replication of the ends of the Mimivirus chromosome in a way similar to the RDR used by T4 (Figure 4). The coiled-coil segment in the phage gp46 protein (homologous to SbcC) is much shorter than in its cellular counterparts. It has been suggested that this may be related to the shorter length of the T4 168 kbp genome and its much denser packing in viral replication centers compared to cellular genomes [4]. Interestingly the length of the predicted coiled-coil segment in R555 is comparable to these of cellular homologs, and appears suitable to bridge two ends of the long Mimivirus genomic DNA (Figure 1). In T4, RecA-type recombinase UvsX has a crucial role in the RDR, but Mimivirus possesses no RecA-type recombinase homolog. Mimivirus B-type DNA polymerase (R322) may be involved in such a recombination process, as the poxvirus B-type DNA polymerase is known to have a recombinase activity [29,30].

Another plausible role of R555 in Mimivirus may be associated with the replication of DNA hairpins. In *E. coli*, the SbcD/SbcC complex has an affinity for DNA hairpin structures (>200 bp stem) and is known to generate DSBs at these sites, which are then repaired by homologous recombination [2,5]. Most Mimivirus genes 3'-UTRs exhibit a palindromic sequence that serves as a polyadenylation site on the transcribed mRNA and tRNA molecules [11,31]. These sequences have the potential of forming hairpin structures (>12 bp stem). Out of the 581 Mimivirus genes for which the 3'transcript ends were mapped, 473 (81.4%) showed these potential hairpin structures [31]. Given their large number of occurrence in the Mimivirus genome, these hairpins, albeit short, may occasionally inhibit DNA replication. R555 might thus be involved in the process ensuring the correct replication of functionally important palindromic sequences.

## Conclusions

Homologs of the Mre11/Rad50 complex play crucial roles in the DSB repair metabolism in cellular organisms. In this study, we showed that Mimivirus R555 gene product corresponds to a fusion of Mre11-like and Rad50-like domains and that its close homologs are specifically abundant in aquatic viruses. We also identified twelve additional cases of similar fusion protein sequences in unrelated cellular organisms as well as in another virus for the first time through a systematic database search. The abundance of the Mre11/Rad50-like fusion genes in viral metagenomes and their sporadic phylogenetic distribution can be explained by recurrent creations of new variants of genes in viruses and their subsequent transfers to different cellular organisms possibly relayed by plasmids or transposons.

## Methods

Mre11/Rad50 and SbcDC fusion proteins were identified by PSI-BLAST [32] using position specific scoring matrices for the COG0419 (SbcC) and COG0420 (SbcD) as queries. Prediction of coiled-coil domains was performed using the Coiled-Coil Prediction Server (http://npsa-pbil.ibcp.fr/; [33]). Multiple sequence alignments were constructed using MAFFT ver. 6 with E-INS-i option [34]. The alignments were examined and columns with more than 50% gaps were trimmed prior to phylogenetic reconstructions. Maximum likelihood phylogenetic analysis was performed using PhyML ver. 3 with the JTT substitution model and 100 bootstrap replicates [35]. We used MEGA5 (http://www.megasoftware.net/; [36]) for tree drawing. The BIOME data set was downloaded from the CAMERA web site [37]. We identified close homologs for the Mimivirus ORFs based on the following procedure [13]. First, all the Mimivirus ORF sequences were compared to the BIOME data set using TBLASTX (E-value <0.1). This initial search resulted into 13,305 metagenomic reads matching to Mimivirus ORFs. These 13,305 sequences were then searched against the UniProt database [38] using BLASTX. This search resulted into 869 metagenomic reads exhibiting their best match to Mimivirus ORFs (E-value <0.1). For each of these 869 sequences, we extracted a segment of the Mimivirus sequence that was aligned with the reads. Next, this partial Mimivirus sequence was searched against the UniProt database (excluding Mimivirus entries in the database) using BLASTP. If the best score obtained from this BLASTP search was lower than the BLASTX score obtained for the alignment of the metagenomic read and Mimivirus sequence, the metagenomic read was kept as a close homolog of the Mimivirus ORF.

## List of abbreviations

DSB: DNA double-strand break; SMC: structural maintenance of chromosomes; APMV: Acanthamoeba polyphaga mimivirus; NCLDV: nucleocytoplasmic large DNA virus; GOS: global ocean sampling; BIOME: metagenomic profiling of nine biomes; HLH: helix-loop-helix; RDR: recombination-dependent DNA replication

## Competing interests

The authors declare that they have no competing interests.

## Authors' contributions

TY conducted the analyses and drafted the manuscript. JCM participated to data interpretation and manuscript drafting. HO designed the experiments, helped the analyses and contributed to manuscript preparation. All authors have read and approved the final manuscript.

## Supplementary Material

Additional file 1 **Mre11/Rad50 fusions mimic known inter-domain interactions**. Crystal structure of the *Thermotoga maritime *Mre11/Rad50 complex (PDB: 3QG5) (a), and multiple sequence alignment of the C-terminal region of Mre11 (SbcD) (b). In (b), red triangles indicate the residue positions of Mre11 suggested being responsible for the direct interaction with Rad50. Click here for file

Additional file 2 **Source of sequences used in the phylogenetic reconstruction**. Sequences were retrieved from NCBI/GenBank, UniProt and KEGG databases. Click here for file

Additional file 3 **Best hit organisms for the ORFs in the GOS-scaffolds encoding a homolog of R555**. (a) ORF based count. 50 of 85 ORFs were most similar to viral sequences. (b) Scaffold based count. Scaffolds having a virus best matching ORF was classified in "virus" category in this figure. 43 of the 68 scaffolds were found to contain at least one viral-like gene. Click here for file

Additional file 4 **List of BLAST best hit for the scaffolds with a homolog of R555**. Database searches were performed against UniProt and RefSeq database. Click here for file

Additional file 5 **Proportion of Mimivirus-like sequences detected in the 87 metagenomes of the BIOME data set**. Green line indicates the level of the percentage of the Mimivirus-like sequences detected in the total BIOME data set. To assess the significance of the number of homologs, we randomly shuffled the sequences in the 87 metagenomic data set using the EMBOSS/SHUFFLE program. Red line indicates the percentage level of Mimivirus-like sequences detected in this artificial data set. Metagenome numbers on the X-axis correspond to the original ID numbers (Table S2 of [15]). Click here for file
