# Supplementary material for: Mimivirus reveals Mre11/Rad50 fusion proteins with a sporadic distribution in eukaryotes, bacteria, viruses and plasmids
Source: Virol J. 2011 Sep 7;8:427. doi: 10.1186/1743-422X-8-427 (PMC3175470; doi:10.1186/1743-422X-8-427)
Supplement: Additional file 1 — Mre11/Rad50 fusions mimic known inter-domain interactions. Crystal structure of the Thermotoga maritime Mre11/Rad50 complex (PDB: 3QG5) (a), and multiple sequence alignment of the C-terminal region of Mre11 (SbcD) (b). In (b), red triangles indicate the residue positions of Mre11 suggested being responsible for the direct interaction with Rad50. [file 1743-422X-8-427-S1.PDF]

(a)

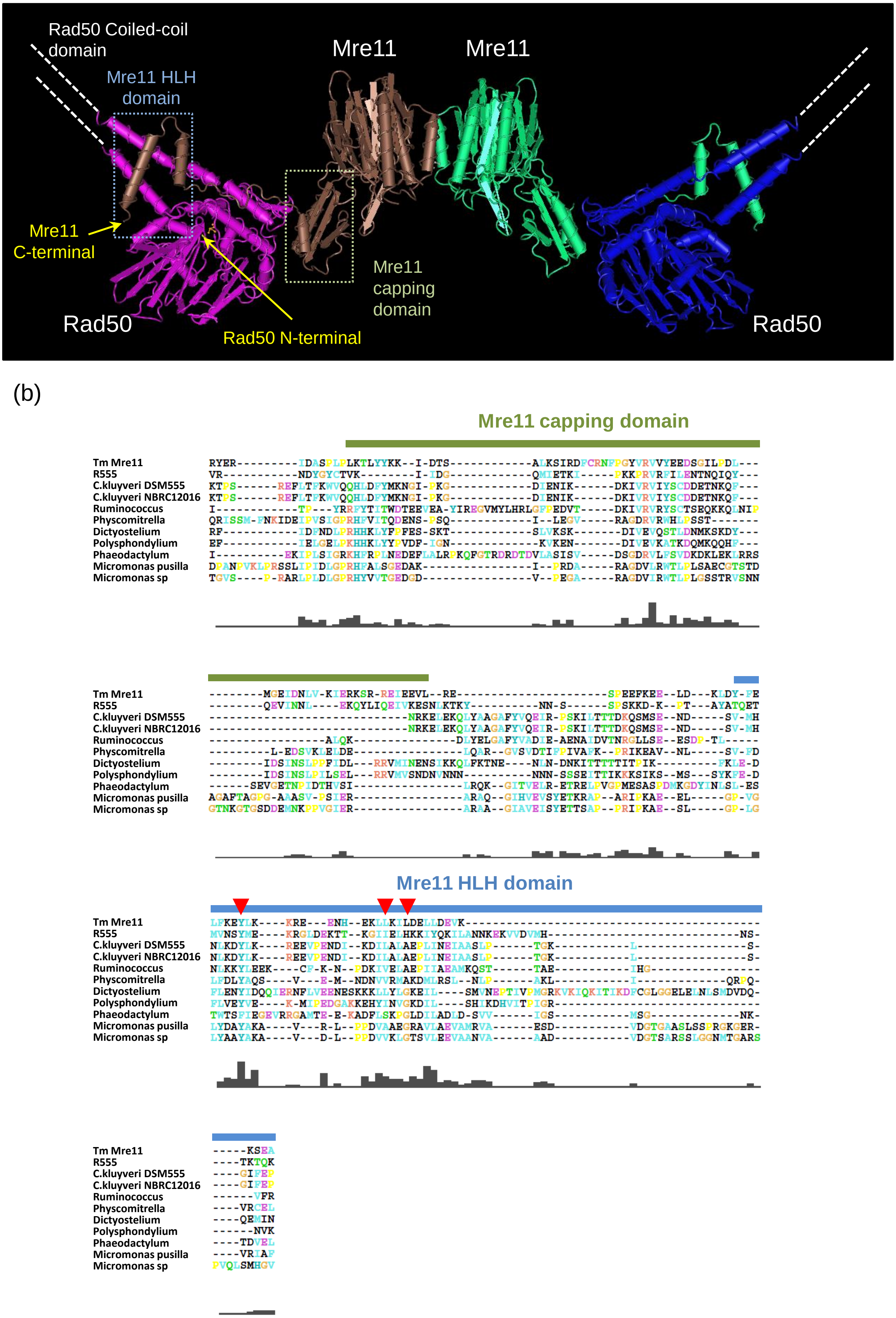

Additional file 1: Mre11/Rad50 fusions mimic known inter-domain interactions . Crystal structure of the Thermotoga maritime Mre11/Rad50 complex (PDB: 3QG5) (a), and multiple sequence alignment of the C-terminal region of Mre11 (SbcD) (b). In (b), red triangles indicate the residue positions of Mre11 suggested being responsible for the direct interaction with Rad50.
