# Supplementary material for: Mimivirus reveals Mre11/Rad50 fusion proteins with a sporadic distribution in eukaryotes, bacteria, viruses and plasmids
Source: Virol J. 2011 Sep 7;8:427. doi: 10.1186/1743-422X-8-427 (PMC3175470; doi:10.1186/1743-422X-8-427)
Supplement: Additional file 3 — Best hit organisms for the ORFs in the GOS-scaffolds encoding a homolog of R555. (a) ORF based count. 50 of 85 ORFs were most similar to viral sequences. (b) Scaffold based count. Scaffolds having a virus best matching ORF was classified in "virus" category in this figure. 43 of the 68 scaffolds were found to contain at least one viral-like gene. [file 1743-422X-8-427-S3.PDF]

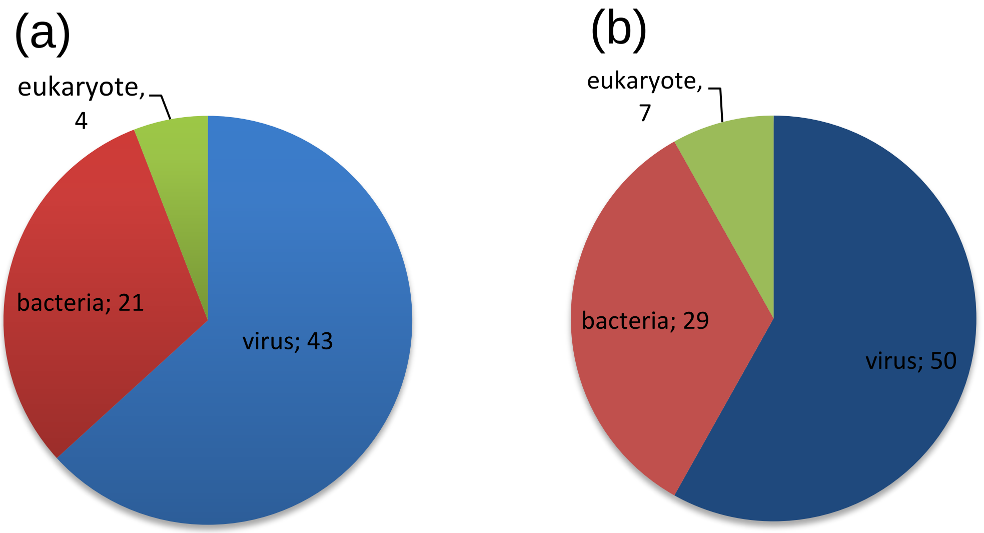

Additional file 3: Best hit organisms for the ORFs in the GOS-scaffolds encoding a homolog of R555. (a) ORF based count. 50 of 85 ORFs were most similar to viral sequences. (b) Scaffold based count. Scaffolds having a virus best matching ORF was classified in "virus" category in this figure. 43 of the 68 scaffolds were found to contain at least one viral-like gene.
