# Supplementary material for: Mimivirus reveals Mre11/Rad50 fusion proteins with a sporadic distribution in eukaryotes, bacteria, viruses and plasmids
Source: Virol J. 2011 Sep 7;8:427. doi: 10.1186/1743-422X-8-427 (PMC3175470; doi:10.1186/1743-422X-8-427)
Supplement: Additional file 5 — Proportion of Mimivirus-like sequences detected in the 87 metagenomes of the BIOME data set. Green line indicates the level of the percentage of the Mimivirus-like sequences detected in the total BIOME data set. To assess the significance of the number of homologs, we randomly shuffled the sequences in the 87 metagenomic data set using the EMBOSS/SHUFFLE program. Red line indicates the percentage level of Mimivirus-like sequences detected in this artificial data set. Metagenome numbers on the X-axis correspond to the original ID numbers (Table S2 of [15]). [file 1743-422X-8-427-S5.PDF]

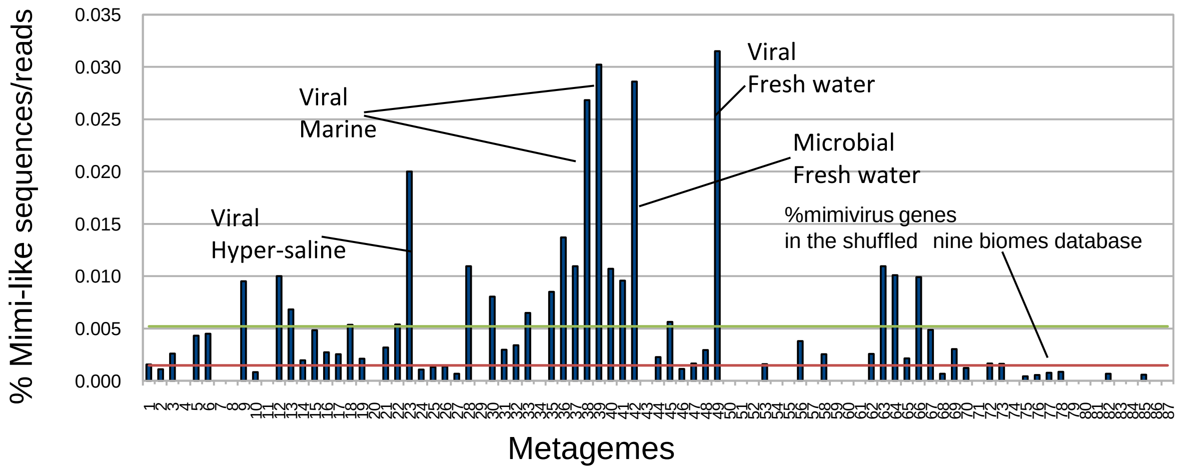

Additional file 5: Proportion of Mimivirus-like sequences detected in the 87 metagenomes of the BIOME data set. Green line indicates the level of the percentage of the Mimivirus-like sequences detected in the total BIOME data set. To assess the significance of the number of homologs, we randomly shuffled the sequences in the 87 metagenomic data set using the EMBOSS/SHUFFLE program. Red line indicates the percentage level of Mimivirus-like sequences detected in this artificial data set. Metagenome number on the X-axis correspond to the original ID numbers (Table S2 of [15]).
